# Modeling the cost-effectiveness of introducing donation after controlled circulatory death for heart transplantation in France

**DOI:** 10.3389/ti.2026.17149

**Published:** 2026-09-09

**Authors:** Jamal Atfeh, Pascale Guerre, Julien Guihaire, Laurent Sebbag, Matteo Pozzi, Laure Huot

**Affiliations:** 1 Hospices Civils de Lyon, Pôle de Santé Publique, Service Recherche et Epidémiologie Cliniques - Evaluation Economique en Santé, Lyon, France; 2 Université Claude Bernard Lyon 1, Research on Healthcare Performance RESHAPE, INSERM U1290, Lyon, France; 3 Université Claude Bernard Lyon 1, Health Systemic Process, EA 4129 Research Unit, Lyon, France; 4 Adult Cardiac Surgery and Transplantation, Marie Lannelongue Hospital, Le Plessis Robinson, France; 5 Inserm UMR-S 999, School of Medicine, University of Paris Saclay, Le Kremlin Bicêtre, France; 6 Hospices Civils de Lyon, Hôpital Louis Pradel, Service Insuffisance Cardiaque Assistance et Transplantation, Lyon, France; 7 Hospices Civils de Lyon, Hôpital Louis Pradel, Service de Chirurgie Cardiaque, Lyon, France

**Keywords:** cost effectiveness, donation after controlled circulatory death (DCD), donor pool expansion, heart transplantation, policy

## Abstract

Heart transplantation following donation after controlled circulatory death (DCD) could alleviate the global shortage of organs. We assessed the cost-effectiveness of this strategy and outlined the factors enabling an efficient DCD heart program. Two strategies were compared: donation after brain death (DBD) using static cold storage vs. DBD + DCD transplantation, where DCD hearts are preserved with a normothermic *ex situ* perfusion system. Costs (€; 2024 value) and outcomes (quality-adjusted life years (QALYs)) were modelled through a 15-year time horizon. Our main assumption was that DCD would increase transplant access without affecting waitlist mortality, with equivalent post-transplant outcomes. With a 20% annual increase in transplant activity, the DBD + DCD strategy resulted in a mean incremental gain of 0.14 QALYs at an additional cost of €20,618 compared with DBD alone. The incremental cost-effectiveness ratio was €146,373 per QALY gained. An 8% relative reduction in waitlist mortality (i.e., four additional lives saved annually among 500 new waitlisted patients) would make the DCD heart program cost-effective at €100,000 per QALY. Lower consumable costs would further improve cost-effectiveness. Decision-makers should consider these findings in light of the disease severity and the persistent shortage of heart donors that could be alleviated with DCD.

## Graphical Abstract

**Figure:**
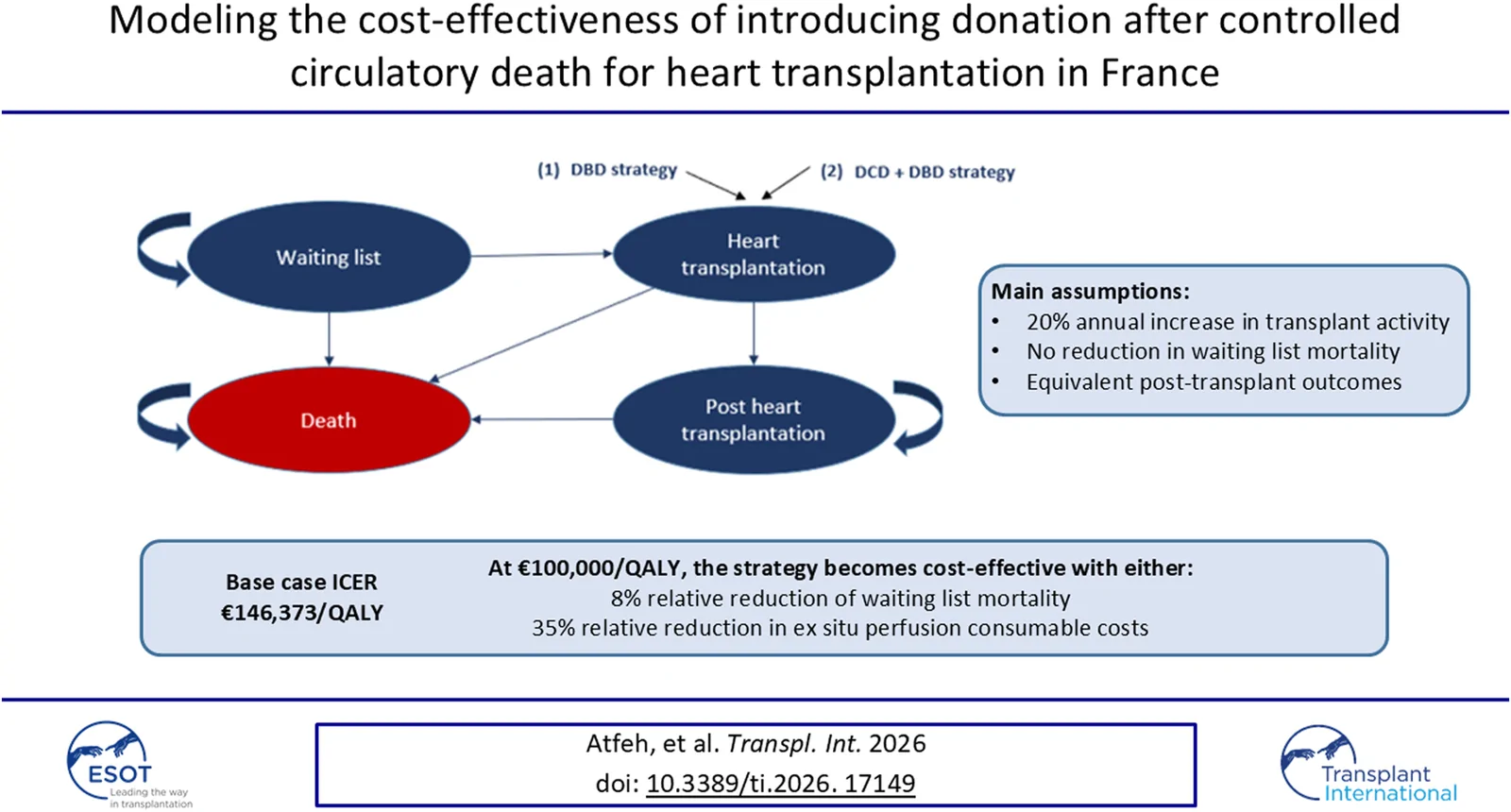

## Introduction

Heart transplantation typically relies on the use of brain-dead donor hearts, which constitutes the main source of donation [1]. However, continuous large imbalances between organ supply and demand for heart transplantations has led to the investigation of new strategies to expand the donor pool [2]. Among these strategies, transplantation with donor hearts after controlled circulatory death has been of particular interest in recent years [3–9]. Increasing consideration of this strategy has been driven by the evolution of technologies, such as *ex situ* organ perfusion systems, allowing for reanimation of the heart after circulatory death and for evaluation of its suitability for transplantation [10]. Heart transplantation using circulatory-dead donor hearts has been proved non-inferior to standard care transplantation using brain-dead donor hearts in terms of risk-adjusted survival at 6 months in a prospective randomized controlled trial [6]. A protocol to assess the feasibility of heart transplantation following Donation after controlled Circulatory Death (DCD) was conducted in France (Protocol PFS20-004, French Agency of Biomedicine) [11]. However, data on the cost-effectiveness of this donor pool expansion strategy are lacking. Indeed, DCD heart procurements are expensive (54,000 euro per normothermic *ex-situ* perfusion consumable) but may improve outcomes for patients with end-stage heart failure by increasing their chances to receive a graft with comparable post-transplantation outcomes [12]. Herein, we sought to model the cost effectiveness of introducing DCD heart transplantation alongside the current standard practice of Donation after Brain Death (DBD) with static cold storage from the French healthcare system perspective and to outline the factors enabling an efficient DCD heart program.

## Materials and methods

A cohort discrete-time state transition model with a time-inhomogeneous Markov process was developed [13]. In these decision models, a hypothetical cohort of individuals transition over discrete time periods (i.e., cycles) between mutually exclusive health states representing the clinical course of the disease [14]. The time inhomogeneous approach relaxes the Markov assumption, allowing transitions, rewards, or both to vary over time [13]. A cost utility analysis framework was used to compare costs and effects (expressed as quality-adjusted life years or QALYs) from the French healthcare system perspective between standard care and the innovative strategy [15]. The model was built and run in R (version 4.2.2) within the R Studio software and using the R package “heemod” [16]. We followed the Consolidated Health Economic Evaluation Reporting Standards (CHEERS) reporting guidelines [17].

### Population

The modelled population consisted of adult patients (age ≥18 years) with end-stage heart failure who were eligible for a first heart transplantation and excluded patients awaiting multi-organ transplantations.

### Comparators

Two strategies were compared. The standard care strategy (DBD strategy) consists of performing transplantations using hearts from brain-dead donors, preserved via static cold storage, which remains the most widespread approach in France. The innovative strategy (DCD + DBD strategy) consists of performing DCD heart transplantations alongside the standard DBD approach. It involves the use of DCD hearts in accordance with the protocol in France (Protocol PFS20-004, French Agency of Biomedicine) [11]. In brief, individuals become donors after controlled withdrawal of life-sustaining treatment, followed by cardiac arrest and circulatory death; the direct procurement procedure is then undertaken, with the heart preserved using a normothermic *ex situ* perfusion system. This approach was developed to introduce heart retrieval within the existing French DCD protocol based on abdominal normothermic regional perfusion (NRP) for abdominal organ procurement.

### Model structure

**FIGURE 1:**
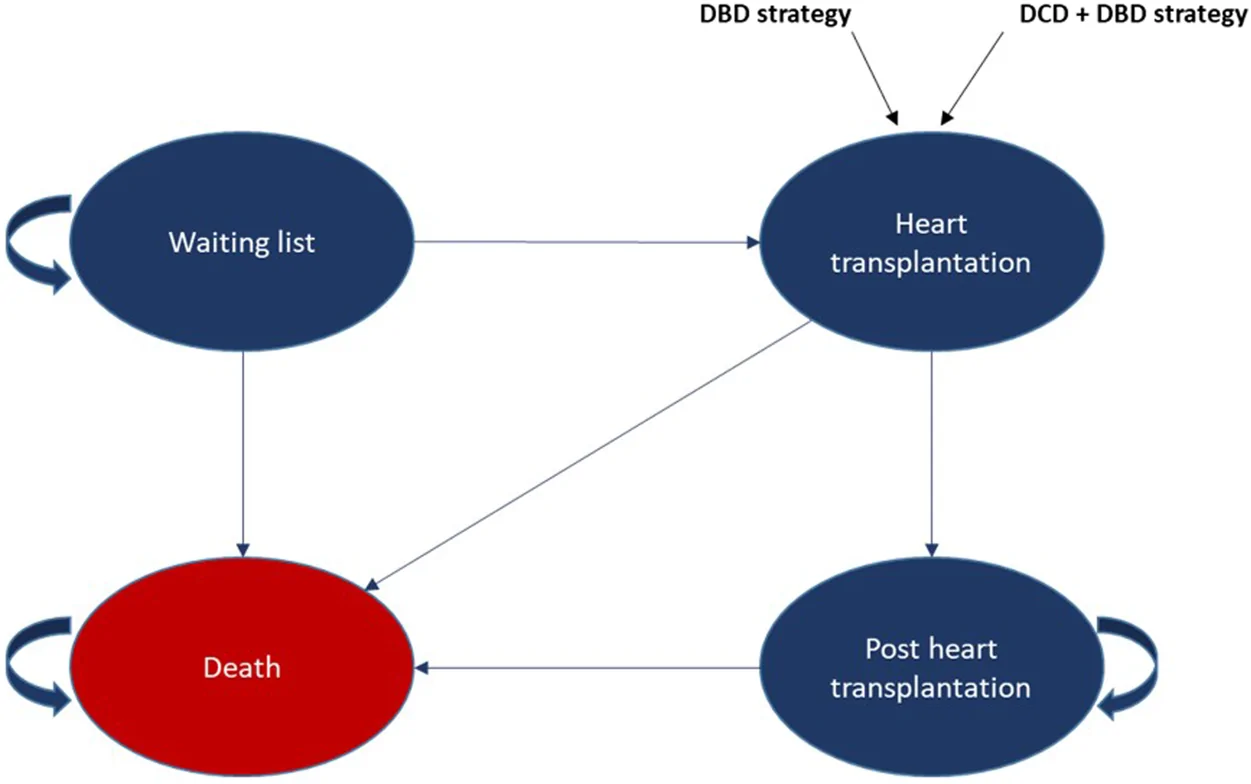
Health state transition diagram. DCD: donation after circulatory death; DBD: donation after brain death. The health state transition diagram consisted of four distinct health states: waiting list, heart transplantation, post heart transplantation, and death. The heart transplantation state is a temporary state that an individual must leave after one cycle. The cycle duration was set to 1 month and time horizon to 15 years.

The model structure was adapted from previously published cost-effectiveness models in end-stage heart failure [18, 19] and in the context of liver transplantation, where strategies aimed at expanding the donor pool were evaluated [20, 21]. It consisted of four distinct health states (Figure 1). The cycle duration was set to 1 month, and half cycle correction was performed using the life table method [22]. A time horizon of 15 years was chosen due to deep uncertainty beyond that period and based on French data from the French Agency of Biomedicine. Most patients are transplanted or deceased within 3 years from registration on the waiting list, and the median survival after transplantation is roughly 12 years [23]. Costs and QALYs were discounted at 2.5% according to French recommendations [24].

### Model parameters and assumptions

Crude epidemiological, cost, and utility estimates used to parametrize the model are presented in Table 1.

**TABLE 1:** Parameters considered in the model.

Parameter	Estimate*	Range*	Distribution**	Source
Transition probabilities	​	​	​	​
Probability (%) of HT at 1-year post listing	61.6	49.3–73.9	Beta	French agency of biomedicine
Probability (%) of death at 1-year post listing	10.2	8.2–12.2	Beta	French agency of biomedicine
Survival after first HT (median in months)	146.7	140.5–152.2	Lognormal	French agency of biomedicine
Annual increase of transplant activity with DCD (%)	20	15–25	Triangle	Jawitz et al, 2020
Costs (€ 2024 price year)	​	​	​	​
Mean cost per patient from WL to 1-year post listing	16,614	10,797–25,771	Gamma	Atfeh et al, 2025
Mean cost per patient of HT surgery with static cold storage	148,958	119,166–178,750	Gamma	DRG 27C054 - French national cost study (2022)
Cost of normothermic *ex-situ* perfusion consumable (including taxes)	54,000	43,200–64,800	Gamma	Unitary purchase price
Mean cost per patient in the first year after HT	13,722	10,695–16,749	Gamma	CUPIDON study (NCT02602691)
Mean cost per patient in the second year after HT	7,625	5,943–9,307	Gamma	CUPIDON study (NCT02602691)
Mean cost per patient in the third year after HT and beyond	3,919	3,054–4,784	Gamma	CUPIDON study (NCT02602691)
Utilities	​	​	​	​
On the waiting list	0.44	0.17–0.71	Beta	Emin et al, 2016
When transplanted	0.74	0.47–1	Beta	Emin et al, 2016
Post-HT	​	​	​	​
First year	0.82	0.64–1	Beta	CUPIDON study (NCT02602691)
1.5 years	0.81	0.63–0.99	Beta	CUPIDON study (NCT02602691)
2 years	0.80	0.62–0.98	Beta	CUPIDON study (NCT02602691)
2.5 years	0.79	0.61–0.97	Beta	CUPIDON study (NCT02602691)
3 years and beyond	0.76	0.58–0.94	Beta	CUPIDON study (NCT02602691)

WL, waiting list; HT, heart transplantation; DCD, donation after controlled circulatory death.

* Estimates and ranges were adjusted to 1-month cycle lengths in the model when necessary. Ranges were used for the deterministic sensitivity analysis.

** Distributions used in the probabilistic sensitivity analysis. Method of moments was used to estimate parameter distributions.

#### Transition probabilities

Short and long-term outcomes after heart transplantation were considered equivalent between the two strategies, consistent with the findings of the DCD Heart Trial (ClinicalTrials.gov number: NCT03831048) and early U.S. experience with a three-year follow-up [6, 25]. In order to assess efficiency in a French context, we mainly used epidemiological and survival data from the French Agency of Biomedicine [23]. For the post-transplantation period, individual patient data were reconstructed from a published survival curve of recipients after a first heart transplantation (period 2004 – June 2022) [26]. We then fitted a log-normal parametric model after comparing the goodness of fit of different models and their plausibility when compared to empirical data and derived monthly probabilities for each model cycle (Supplementary Figures 1a–c; Supplementary Table 1). As no published data exists on the potential of DCD to increase the French heart-donor pool, an estimate based on an analysis of the United Network for Organ Sharing (UNOS) deceased donor database 2005–2014 was used [27]. The study reports a 30% projected annual increase with DCD heart transplantation but acknowledges possible overestimation, as some DCD hearts may be deemed unsuitable after functional assessment. A more conservative 20% annual increase was therefore modeled. This assumption was also considered consistent with the French transplantation context, where DCD heart transplantation is expected to begin with a pilot phase and to be progressively implemented across heart transplant centers. Uncertainty around this parameter was explored in the deterministic sensitivity analysis, using a 15%–25% range.

While the increase of available grafts should improve the probability of transplantation in waitlisted candidates, it is unclear whether expanding the donor pool with DCD would reduce waiting list mortality [12, 28]. Therefore, the assumption made would be conservative in the main analysis and implementing DCD would only increase transplant probability without affecting mortality (worst case scenario).

#### Costs

Given the scarcity of economic information available for outpatient care in this indication, the costs considered were limited to those in a hospital setting, which were supposed to be the main cost drivers. Costs were valued from the healthcare system perspective according to French guidelines, which focuses solely on healthcare production and accounts for all monetary costs of healthcare, regardless of who bears the cost [24, 29]. For the pre-transplantation period, costs were extracted from a previous work which assessed the economic burden of patients awaiting heart transplantation [30]. The cost of DBD transplantation in standard care was based on the Diagnosis Related Group 27C054 and was valued using the French National Cost Study, which produces the closest valuation to the hospital production cost [24]. The cost of DCD transplantation was computed as that of DBD transplantation, plus the cost of using an *ex-situ* perfusion system; its cost being the unit purchase price of consumables of €54,000 including taxes (under the manufacturer’s business model, the equipment was provided free of charge). The total cost of heart transplantation for the innovative strategy was then calculated in proportion to the annual increase in the volume of heart transplantations performed and the ratio between DBD and DCD heart transplantations. For the post-transplantation period, cost data were extracted from the French CUPIDON study [31] (ClinicalTrials.gov identifier NCT02602691), covering individual patient costs from 6 to 36 months after transplantation. The first 6 months were extrapolated from the monthly costs between months 6 and 12 reported in the CUPIDON study. They were also assumed equivalent between the two strategies, in line with the clinical assumptions, and to remain stable beyond 36 months, consistent with published cost-effectiveness models in this indication [18, 19]. All costs were expressed in euros (to convert to US dollars, multiply by 1.16) at 2024 price year and adjusted for inflation based on the French National Institute of Statistics and Economic Studies (INSEE) Consumer Price Indices of the healthcare products and services [32].

#### Utilities

After conducting a literature search, we retrieved a systematic review of health state utilities in patients with heart failure [33]. As no data were available in France, utility scores on the waiting list and at the time of transplantation were extracted from a cross-sectional survey of the United Kingdom Cardiothoracic Transplant Audit with administration of EQ-5D questionnaires [34]. We chose this study because it was the only one to provide utility scores on a population of end-stage heart failure patients eligible for transplantation, defined on the basis of registration on the waiting list. Although cross-country differences in general population preferences may exist, France and the United Kingdom are culturally and economically comparable; therefore, UK-based utilities were considered reasonable proxies for France. For the DCD + DBD strategy, in the base case analysis, no utility decrement was applied to the temporary transplantation state in relation to the potential increased risk of primary graft dysfunction (PGD) with circulatory-dead donor hearts [35]. It was expected to be marginal given the ratio between DBD and DCD heart transplantations and the relative frequency of these adverse events [36, 37]. A worst-case scenario related to PGD was tested further. Utility scores after heart transplantation were extracted from the French CUPIDON study. To obtain the number of QALYs, utility scores were multiplied by the number (or fractions) of life years gained.

### Analyses

A deterministic base-case analysis was performed using the point estimates of each model parameter. Model outputs were computed to an incremental cost-effectiveness ratio (ICER) expressed as a cost per QALY gained (€/QALY).

A univariate deterministic sensitivity analysis (DSA) was performed in order to test the robustness of the estimation of our ICER. Parameter values were varied one at a time based on the input’s source (95% confidence interval or standard error) or set to a specific range if not available (Table 1). Results of the DSA were presented in a tornado diagram. Scenario analyses were performed to explore the impact of certain model settings and assumptions. Scenarios 1 and 2 tested the application of a discount rate of 4.5% and 0% respectively, according to French guidelines [24]. Scenario 3 modeled a 10% disutility and a 20% cost increase (based on expert opinion) to the heart transplantation state in the DCD + DBD strategy to reflect a pessimistic PGD assumption with DCD, potentially affecting both costs and patient outcomes significantly. Scenario 4 explored the impact of an alternative, more pessimistic, but still fairly well fitted parametric survival distribution (i.e., gamma). Finally, different time horizons were assessed in scenario 5 and 6 (respectively 10 and 20 years).

A probabilistic sensitivity analysis (PSA), running 1000 Monte Carlo simulations, was performed to account for statistical uncertainty. Distributions were assigned according to the type of parameter. The PSA results were presented on a cost-effectiveness plane and using a cost-effectiveness acceptability curve to represent the probability that the innovative strategy is cost-effective according to different willingness-to-pay (WTP) thresholds (using the R package “BCEA” [38]).

Finally, two independent threshold analyses were performed to assess, holding all other parameters constant: (1) the required change in waiting list mortality with DCD implementation for the innovative strategy to be cost-effective at given thresholds and (2) the reduction in consumable costs for the innovative strategy to be cost-effective at given thresholds.

### Model validation

Face validity of the model (structure, inputs, and outputs) and assumptions were assessed by health economists and clinical experts in heart transplantation. Internal validation included structured debugging, extreme value testing, and verification of all calculations. Markov traces are presented in Supplementary Figure 3.

## Results

**FIGURE 2:**
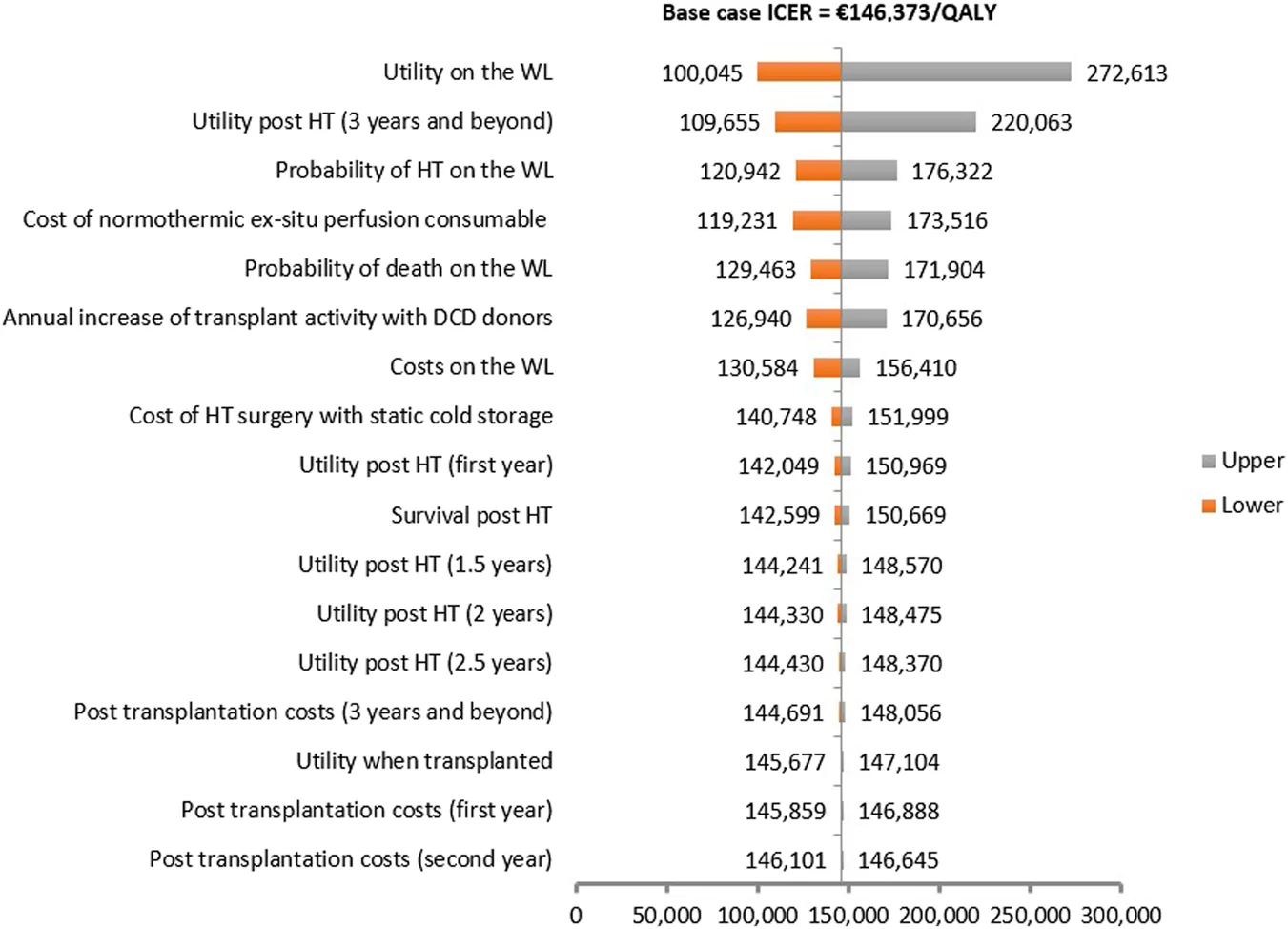
Results from the univariate deterministic sensitivity analysis–Tornado diagram. WL, waiting list; HT, heart transplantation; DCD, donation after circulatory death; QALY, quality-adjusted life year; ICER, incremental cost effectiveness ratio. The tornado diagram illustrates the impact of varying model parameters (one by one) on the incremental cost-effectiveness ratio.

Over a 15-year time horizon, patients in the DBD strategy experienced 5.53 QALYs at a cost of €184,110 whereas patients in the DCD + DBD strategy experienced 5.67 QALYs at a cost of €204,728. These outcomes resulted in an incremental gain of 0.14 QALYs at an incremental cost of €20,618, producing an ICER of €146,373 per QALY gained. The results of the DSA are presented in Figure 2. The main drivers of the ICER estimate were the utility of patients on the waiting list and after 3 years post-transplantation. Results from the scenario analyses were reported in Table 2. The application of a utility decrement and an increase in the cost of heart transplantation in a pessimistic scenario of major impact of PGD with DCD resulted in an ICER of €180,763 per QALY at 15 years.

**TABLE 2:** Results from the scenario analyses.

Scenario	Incremental cost (€)	Incremental QALY	ICER (€/QALY)
Base case	20,618	0.14	146,373
Scenario 1: 4.5% discount rate applied	20,651	0.13	162,133
Scenario 2: No discount rate applied	20,588	0.16	127,015
Scenario 3: Increased primary graft failure impact	24,441	0.13	180,763
Scenario 4: Gamma distribution for post-transplant survival	20,622	0.14	147,246
Scenario 5: 10-year time horizon	20,440	0.11	192,933
Scenario 6: 20-year time horizon	20,775	0.17	121,462

QALY, quality-adjusted life year; ICER, incremental cost effectiveness ratio.

**FIGURE 3:**
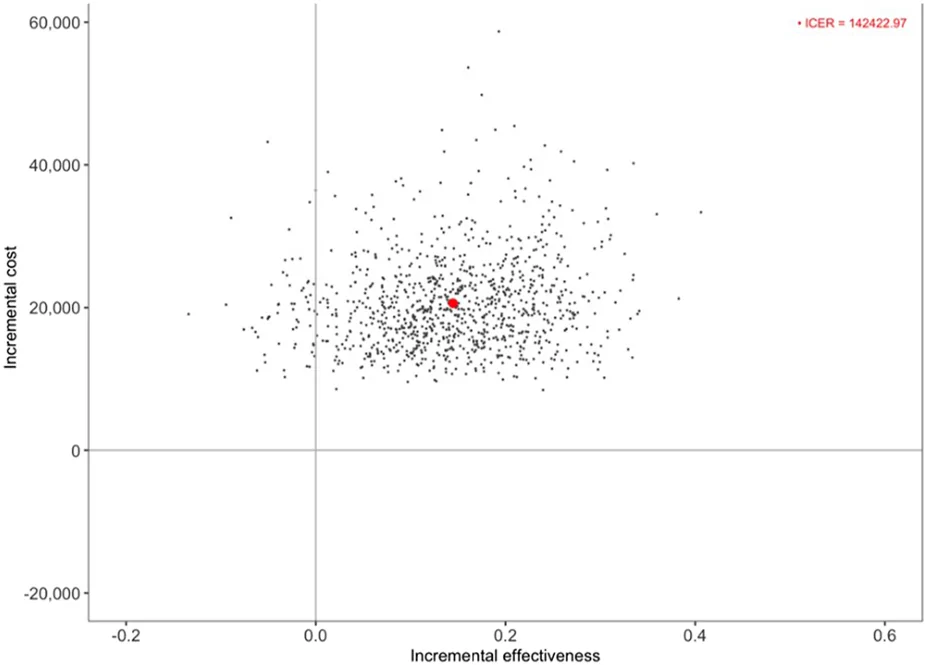
Results from the probabilistic sensitivity analysis - Cost-effectiveness plane. ICER: incremental cost effectiveness ratio. Each point represents a simulated ICER from the probabilistic sensitivity analysis (1000 simulations). The mean probabilistic ICER was €142,423 per QALY.

**FIGURE 4:**
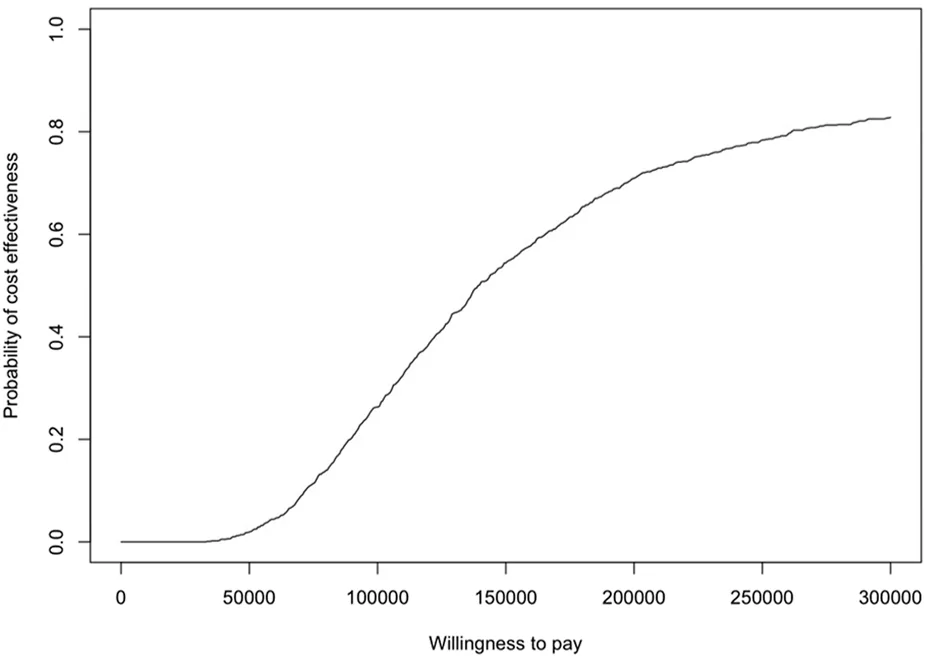
Results from the probabilistic sensitivity analysis - Cost-effectiveness acceptability curve. The cost-effectiveness acceptability curve represents the probability of cost-effectiveness according to different willingness-to-pay thresholds. The innovative strategy reached 50% and 80% chances of being cost-effective, respectively, at a willingness to pay of €139,200 and €261,000 per QALY.

Results of the PSA are presented in Figures 3, 4. The mean probabilistic ICER of the DCD + DBD strategy compared to DBD alone was €142,423 per QALY with a mean incremental cost of €20,609 (95% credible interval: €11,220–€37,453) and a mean incremental QALY gain of 0.14 (95% credible interval: –0.03–0.3). The innovative strategy reached 50% and 80% chances of being cost-effective respectively at a WTP of €139,200 and €261,000 per QALY.

**FIGURE 5:**
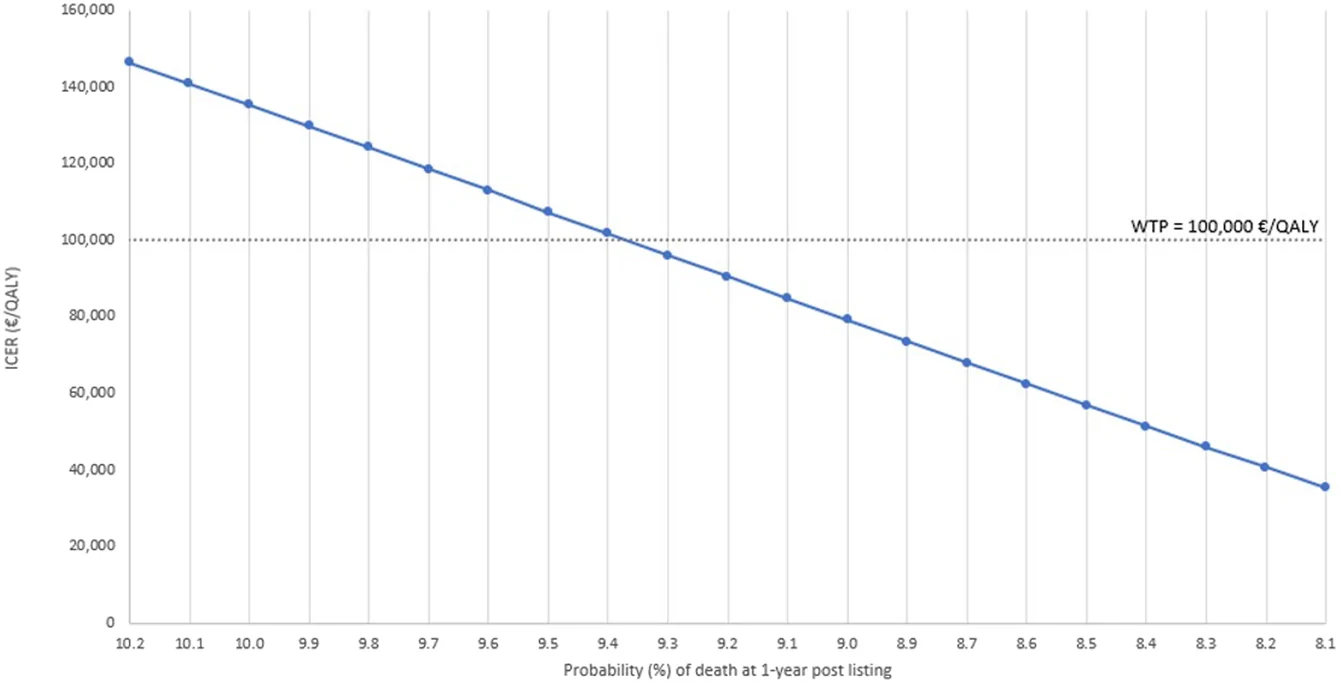
Results from the threshold analysis - Waiting list mortality. The figure shows, holding all other parameters constant, the required change in waiting list mortality with DCD implementation for the innovative strategy to be cost-effective at given willingness-to-pay thresholds. For example, taking a willingness-to-pay of €100,000 per QALY, the DCD + DBD strategy is cost-effective if a 0.8% reduction in waiting list mortality is reached with the donor pool expansion (i.e., moving from 10.2% to 9.4% probability of death 1 year after listing).

**FIGURE 6:**
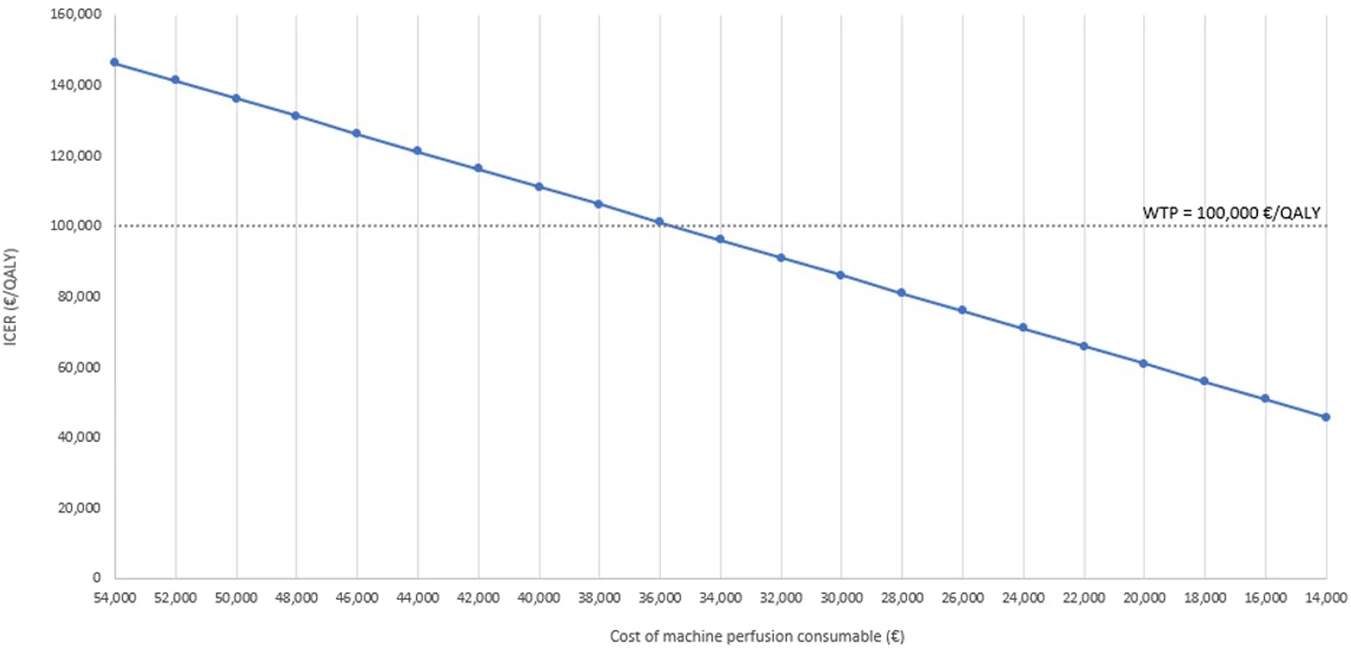
Results from the threshold analysis - Machine perfusion consumable costs. The figure shows, holding all other parameters constant, the required reduction in consumable costs for the innovative strategy to be cost-effective at given willingness-to-pay thresholds. For example, taking a willingness-to-pay of €100,000 per QALY, the DCD + DBD strategy is cost-effective if a 35% reduction in consumables costs is negotiated (i.e., moving from €54,000 to €35,500 per consumable).

The independent threshold analyses are presented in Figures 5, 6 and can be interpreted according to different willingness-to-pay thresholds. For example, taking a WTP of €100,000 per QALY, the DCD + DBD strategy is cost-effective if (1) an 8% relative reduction in waiting list mortality is reached with the donor pool expansion (i.e. 0.8% absolute reduction, from 10.2% to 9.4% probability of death 1 year after listing) and (2) a 35% relative reduction in consumables costs is negotiated (i.e., moving from €54,000 to €35,500 per consumable).

## Discussion

To our knowledge, this is the first study assessing the cost-effectiveness of implementing heart transplantation following DCD, according to a protocol with direct procurement procedure (i.e., involving an *ex-situ* perfusion machine).

The assumptions underlying our model structure and parameters are primarily drawn from the existing literature in this area: our main hypothesis is that the introduction of DCD heart transplantations could increase the odds of a candidate to receive a graft [12, 28], while achieving outcomes comparable to standard care transplantation [3–6].

These assumptions are further supported by recent international experience, including long-term Australian data showing comparable post-transplant survival between DCD and DBD recipients up to 10 years using direct procurement followed by *ex-situ* machine perfusion [39].

For aspects where evidence was limited or uncertain, we adopted a conservative approach in our assumptions. In particular, we assumed that an increase in the supply of donors would not affect the mortality risk on the waiting list in the base case analysis. Sensitivity analyses were also performed. We specifically tested a pessimistic alternative scenario in which PGD, a commonly feared complication with DCD [36, 37], had a major impact on costs and quality of life during the heart transplantation state in the DCD + DBD strategy, which led to an ICER of €180,763 per QALY gained over 15 years. Also, if implementing DCD heart transplantations were to reduce waiting list mortality, the strategy would offer greater value for money. In our model, an 8% relative reduction of probability of death 1 year after listing corresponded to an ICER of €100,000 per QALY. In France, an average of 500 patients are newly registered on the waiting list each year, which would translate to approximately four additional lives saved per year for the DCD program to be cost-effective at this willingness-to-pay threshold. Additionally, lowering consumable costs could further improve the cost-effectiveness of DCD heart transplantation.

This health economic evaluation aims to provide valuable insights for decision-making in countries where DCD heart transplantation procedures are not yet authorized and/or reimbursed. In some countries such as France, no predefined cost-effectiveness threshold is yet used as a formal decision-making criterion [40]. In practice, decisions are often influenced implicitly by both the cost-effectiveness ratio and the clinical severity or burden associated with the condition [41]. End-stage heart failure is an advanced stage of disease known to be associated with poor prognosis and impaired quality of life [34, 42, 43]. Given the persistent and worldwide shortage of heart donors [44], it may be regarded as falling within the scope of unmet (or partially unmet) medical needs [45]. These aspects therefore also need to be considered in the decision-making process. To help the decision, threshold analyses were also provided. They could serve as a foundation for value-based negotiations between decision-makers and manufacturers to support fair (yet sustainable) pricing of machine perfusion consumables. They could also help align heart transplantation practices with value-based objectives for efficient DCD heart programs, including reducing waiting list mortality, as this parameter appears to be pivotal for the cost-effectiveness of the strategy.

Direct procurement followed by *ex-situ* machine perfusion is not the only approach currently used for DCD heart transplantation [1]. Alternative strategies, particularly thoraco-abdominal NRP followed by static cold storage, have demonstrated favorable outcomes, notably in the recent Spanish national experience [8].

Direct procurement followed by *ex-situ* machine perfusion was considered in the present analysis as this approach was developed to introduce heart retrieval within the existing French DCD protocol based on abdominal NRP for abdominal organ procurement [11]. Therefore, it should be acknowledged that our findings are specific to this procedure, reflecting the French setting, and should not be extrapolated to strategies based on thoraco-abdominal NRP, which involve different procurement procedures and resource requirements.

Nevertheless, from an international perspective, countries such as Australia, the United States, and the United Kingdom initially implemented direct procurement followed by *ex-situ* machine perfusion for DCD hearts without combining heart procurement with abdominal NRP. Considering the favorable outcomes associated with abdominal-NRP for liver and kidney transplantations in the French experience [46, 47], recent efforts to promote the international standardization of DCD practices involving the use of NRP [48], and the health economic considerations outlined in the present analysis, our findings may provide useful information for healthcare systems considering the integration of abdominal NRP alongside direct heart procurement and *ex-situ* machine perfusion.

The main strength of this study was its use of epidemiological and survival data from the French organ transplantation agency to parametrize the model. Also, individual patient cost data in a French context were used as model inputs, retrieved from a cost of illness study and a randomized controlled trial with cost data collection (i.e., they were not reconstituted based on practice recommendations). Economic models are, by definition, simplifications of the reality, but we implemented a time-inhomogeneous Markov process to the decision model to help reflect the clinical course of the disease and associated costs and outcomes. Moreover, the model could be adapted to support the evaluation of DCD implementation in different healthcare settings globally.

Our study does have limitations. One limitation is that we could not perform subgroup analyses based on patient characteristics and pathways, which would have been interesting given the inherent heterogeneity of this population [18]. Due to the scarcity of data in this indication, we had to assume the candidate population at the entry of the model as homogenous. It might be possible that specific populations with different levels of graft priority slightly differ in terms of transplantation access, cost, and consequences. The analysis was limited to hospital costs and may therefore underestimate total costs, although hospital care was assumed to be the main cost driver in this indication. Additional implementation costs related to logistics, coordination, and learning curves were not explicitly modeled due to limited available data, but uncertainty related to procedure costs was partially explored in the DSA. Indeed, these costs remain difficult to estimate accurately in the French setting, given the very recent authorization of DCD heart transplantation in the country (March 2026). Accurately estimating these additional costs would require dedicated prospective micro-costing analyses of the procedure, which were beyond the scope of this early economic evaluation. However, we assumed that DCD heart procurement would not adversely affect abdominal organ procurement, consistent with the recently reported French national feasibility study, in which adding heart retrieval in the setting of abdominal NRP did not compromise the procurement of other organs [11]. Another limit is the absence of utility estimates on the pre-transplant period and after 3 years in a French context, which would be interesting to collect as being main drivers of uncertainty in the DSA.

Finally, in the present model, the impact of donor pool expansion was captured among listed candidates. However, increasing the availability of donor hearts may affect listing practices and outcomes in patients with advanced heart failure who are currently not listed because of persistent donor shortage. These broader effects were not modelled due to their inherent uncertainty but may further improve the overall benefits associated with DCD heart transplantation.

Future research should aim to evaluate the potential change of the candidates’ waiting list mortality risk with the implementation of DCD procedures. Organizational aspects (perfusionists, coordination of extended heart procurement, etc.) should also be investigated in real-world settings. A budget impact analysis could be of interest to assess the sustainability of this strategy for healthcare payers.

In conclusion, our results suggest that, under the assumption of a 20% annual increase in transplant activity—improving the odds of a candidate receiving a graft without affecting waitlist mortality and assuming equivalent post-transplant outcomes—implementing DCD heart transplantation alongside DBD would result in an ICER of €146,373 per QALY gained over a 15-year time horizon from the French healthcare system perspective. Lowering consumable costs and reducing waitlist mortality are key factors enabling an efficient DCD heart program.

## Data Availability

The raw data supporting the conclusions of this article will be made available by the authors, without undue reservation.
