# Supplementary material for: Modeling the cost-effectiveness of introducing donation after controlled circulatory death for heart transplantation in France
Source: Transpl Int. 2026 Sep 9;39:17149. doi: 10.3389/ti.2026.17149 (PMC13597464; doi:10.3389/ti.2026.17149)
Supplement: Supplementary file 1 [file Supplementaryfile1.docx]

Supplementary Material

**eFigure 1a** Survival curve used for the post heart transplantation states, based on published data from the French Agency of Biomedicine

**eFigure 1b** **Estimated hazard function** for the post heart transplantation survival

**eFigure 1c** Estimated survival curves for different parametric distributions (post heart transplantation survival)

**eTable 1** Summary of Akaike Information Criterion and Bayesian Information Criterion scores

**eFigure 2** Markov traces

**eFigure 1a** Survival curve used for the post heart transplantation states, based on published data from the French Agency of Biomedicine *(Agence de la Biomédecine. Rapport médical et scientifique. Saint-Denis La Plaine. 2023)*

Time in months

*.*

**eFigure 2b** **Estimated hazard function for the post heart transplantation survival**

Time in months

**eFigure 2c** Estimated survival curves for different parametric distributions (post heart transplantation survival)

Time in months

**eTable 1**: Summary of Akaike Information Criterion and Bayesian Information Criterion scores

| **Distribution** | | **Akaike Information Criterion** | | **Bayesian Information Criterion** |
| --- | --- | --- | --- | --- |
| Lognormal | 29146.2636 | | 29159.9062 | |
| Log-logistic | 29306.7 | | 29320.3426 | |
| Weibull | 29310.0688 | | 29323.7113 | |
| Gamma | 29333.7721 | | 29347.4147 | |
| Gompertz | 32163.8792 | | 32177.5218 | |
| Exponential | 33418.7358 | | 33425.5571 | |

**eFigure 3** Markov traces per state and strategy

DCD: donation after circulatory death

DBD: donation after brain death

HT: heart transplantation
